# The Measurement of Subjective Value and Its Relation to Contingent Valuation and Environmental Public Goods

**DOI:** 10.1371/journal.pone.0132842

**Published:** 2015-07-29

**Authors:** Mel W. Khaw, Denise A. Grab, Michael A. Livermore, Christian A. Vossler, Paul W. Glimcher

**Affiliations:** 1 Center for Neural Science, New York University, New York City, New York, United States of America; 2 Institute for Policy Integrity, New York University School of Law, New York City, New York, United States of America; 3 School of Law, University of Virginia, Charlottesville, Virginia, United States of America; 4 Department of Economics and Howard H. Baker Jr. Center for Public Policy, University of Tennessee, Knoxville, Tennessee, United States of America; University of Vienna, AUSTRIA

## Abstract

Environmental public goods—including national parks, clean air/water, and ecosystem services—provide substantial benefits on a global scale. These goods have unique characteristics in that they are typically “nonmarket” goods, with values from both use and passive use that accrue to a large number of individuals both in current and future generations. In this study, we test the hypothesis that neural signals in areas correlated with subjective valuations for essentially all other previously studied categories of goods (ventromedial prefrontal cortex and ventral striatum) also correlate with environmental valuations. We use contingent valuation (CV) as our behavioral tool for measuring valuations of environmental public goods. CV is a standard stated preference approach that presents survey respondents with information on an issue and asks questions that help policymakers determine how much citizens are willing to pay for a public good or policy. We scanned human subjects while they viewed environmental proposals, along with three other classes of goods. The presentation of all four classes of goods yielded robust and similar patterns of temporally synchronized brain activation within *attentional *networks. The activations associated with the traditional classes of goods replicate previous correlations between neural activity in valuation areas and behavioral preferences. In contrast, CV-elicited values for environmental proposals did not correlate with brain activity at either the individual or population level. For a sub-population of participants, CV-elicited values were correlated with activity within the dorsomedial prefrontal cortex, a region associated with cognitive control and shifting decision strategies. The results show that neural activity associated with the subjective valuation of environmental proposals differs profoundly from the neural activity associated with previously examined goods and preference measures.

## Introduction

Environmental public goods—including national parks, clean air, and ecosystem services—provide substantial benefits on a global scale [1] and are the subject of organizational and individual decisions. Environmental public goods are typically “nonmarket” goods, with values that accrue to a large number of individuals both in current and future generations. Oftentimes, the dominant source of these values is tied to passive use, such as the existence value of a scenic resource. To estimate the benefits associated with environmental goods at a behavioral level, the most widely used approach is contingent valuation (CV): a stated preference, survey-based approach that elicits the willingness to pay for (or accept) proposed changes in the provision of public goods [2]. CV is the only established approach for estimating passive-use values, and it is frequently used in the context of government benefit-cost analysis as well as in litigation over natural resource damages.

As a *stated preference* approach, the CV procedure presents survey respondents with information on a nonmarket good (often a potential public policy to provide an environmental good such as a national park) and asks questions that attempt to reveal the monetary values people place on these goods. This approach contrasts with *revealed preference* methods, which determine values based on observed and impactful behaviors, such as market transactions or consumer choices. Since revealed preference methods are tied to observable behaviors, it is not possible to use these methods for estimating passive-use values. These values, such as the existence value of a resource, represent a large component of the total value of many environmental public goods. Thus, the measurement of stated preferences is warranted in the assessment of total economic values of public policies and assets [3]. Indeed, CV has been used in the National Resource Damage Assessment process to determine the extent of resource injuries after major environmental events, such as *Exxon Valdez*’s 11-million-gallon oil spill in Alaska’s Prince William Sound. For such issues, stated preference surveys—most often in the form of CV—represent the only established approach to estimating the economic value at stake. It should be noted that despite its wide use, there is ongoing debate over the validity of the CV method for environmental goods [4–6].

Across disciplinary lines, neurobiologists using functional magnetic resonance imaging (fMRI) techniques have discovered robust correlations between the values people place on goods and the blood-oxygen-level-dependent (BOLD) signal at two specific loci. These correlations ubiquitously connect brain activity within the ventromedial prefrontal cortex (vmPFC) and ventral striatum (VS) with estimates of how subjects behaviorally value goods using a wide range of preference-elicitation methods [7,8]. Notably, these kinds of correlations have been observed for a variety of good types and with a wide variety of behavioral methods for assessing value. Correlations between brain activity in these areas and behaviorally elicited valuations have been observed for revealed and stated preference studies involving food items [9–11], ratings toward daily activities [12], revealed preferences over charitable donations [13–16], ethical decision making [17], and even in laboratory economics experiments involving the private provision of public goods [18] Such findings have even led to the hypothesis that *all* human valuations may be reflected in activity that can be measured in these two brain areas [19–21]. In support of this hypothesis, meta-analyses of brain imaging data [22,23] have demonstrated that activity in these two brain regions is a ubiquitous feature of *all* forms of valuation that have so far been studied. However, the neural representation of subjective values for environmental public goods has not been established. Further, the unique characteristics of these goods—such as the previously mentioned passive use component, and their status as non-marketed items—prevent obvious extrapolation from existing datasets. Here we compare the neural representation of subjective values for four classes of goods, including a set of environmental public goods behaviorally valued with a standard CV procedure.

One goal of this study is to determine whether the CV procedure—when used to value environmental proposals—also yields preferences that correlate with brain activity in established valuation areas. Put another way, all previously tested valuation procedures are associated with brain activation in the vmPFC and VS when subjects considered, valued, or chose between previously tested types of goods. Thus, we aim to compare this classical result with the subjective valuation of environmental goods and any of its associated neural correlates. We find that all four classes of goods examined yielded robust trial-related activation within attentional networks. Replicating previous studies, we also find that the average vmPFC and VS BOLD activations are associated differentially with the most preferred and least preferred goods for each of the traditional goods classes. Robust and replicated correlations are observed for the values individuals placed on daily activities, snack foods, and consumer goods. However, we find no evidence of a relationship between brain activity in these areas and CV-elicited valuations of environmental goods. Puzzlingly, we find that the neural activations elicited by the environmental goods in these areas are lower than the average BOLD activity elicited by snack foods. This is true despite the fact that the average monetary valuations elicited by the CV procedure for the environmental goods are greater than the valuations reported by our subjects for snack foods. These findings suggest that environmental valuations (as measured by CV) are associated differently to neural activity than previously examined valuation procedures and goods.

## Methods

This study was approved under the New York University Institutional Review Board University Committee on Activities Involving Human Subjects IRB# 13–9417. This submission was reviewed and approved following an Expedited Review at 45 CFR 46 110(b)[1] Categories 4 and 7. All subjects provided written consent before participating.

Thirty subjects (16 female, mean age = 23.78) participated in a two-stage experiment with three goals aimed at allowing us to compare brain activations across types of goods and valuation procedures. Our first goal was to replicate three separate fMRI valuation studies to demonstrate that our procedures, subjects, and measured patterns of neural activity were well aligned with previous work and to provide a direct basis for comparison with the environmental proposals’ CV data. Second, we sought to measure the general pattern of brain activation associated with viewing environmental proposals. Third, we sought to test whether valuations for environmental goods are correlated with brain activations in areas specifically associated, in previous studies, with subjective valuation.

**Fig 1:**
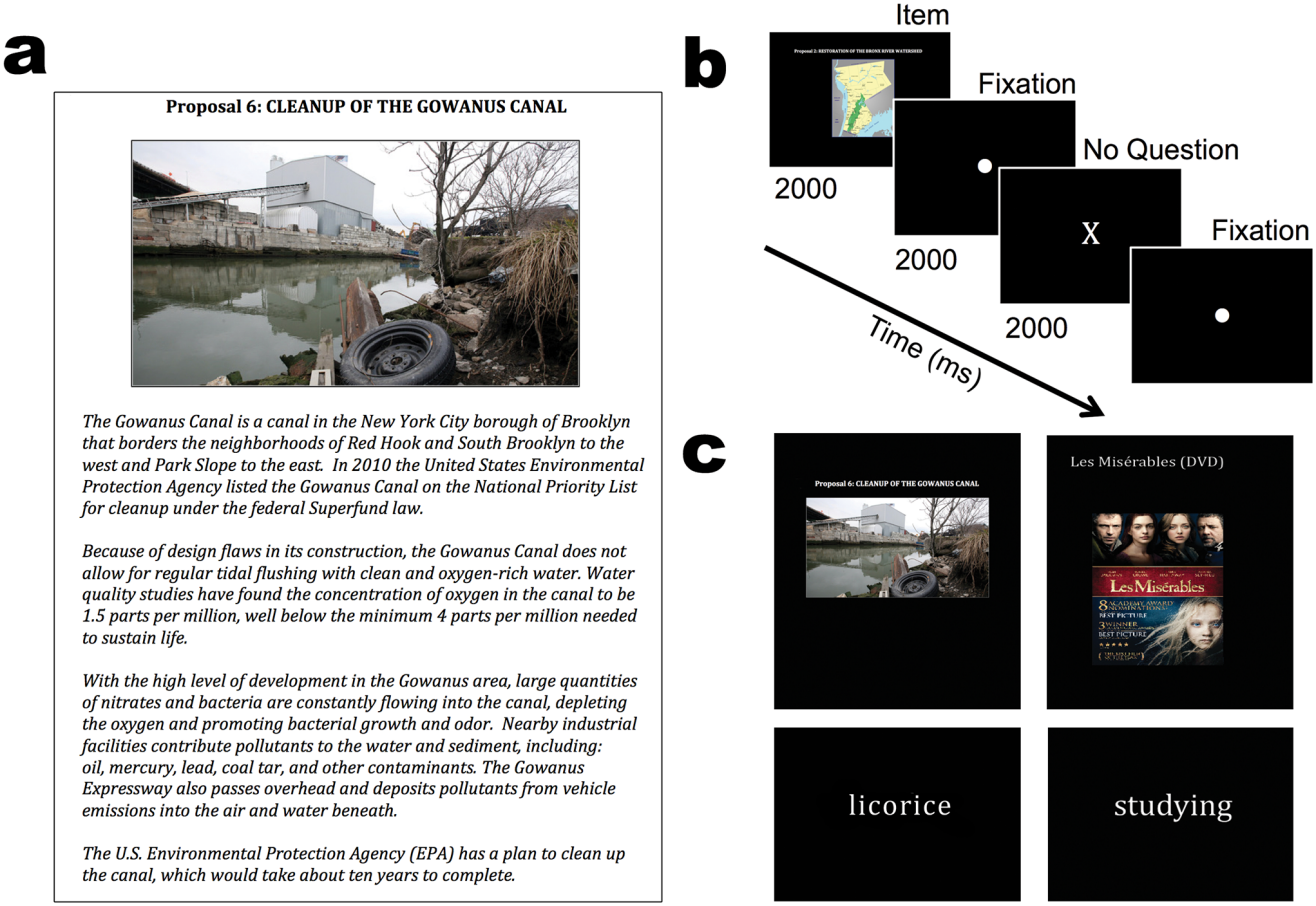
(a) Sample page from 1 (out of 6) environmental proposals that were designed for the experiment. (b) Timeline of a trial inside the scanner, whereby subjects witnessed each good for 2 s, followed by a fixation point for 2 s. Subjects then either saw a question trial for alertness or a cross lasting for 2 s. A fixation point was then displayed for 6, 8, or 10 s chosen with uniform probability. (c) Different good types presented centered on the screen during the scanning session. Clockwise from top left: environmental proposals, consumer goods, daily activities, and snack items.

In the first stage of our experiment, subjects in the scanner viewed four classes of goods in a randomly interleaved order: snack foods, consumer goods, daily activities, and environmental proposals. Following the procedure by Levy et al. [7], subjects were instructed to think about the value of the item they saw on-screen, in dollar terms (or in the event of an activity being presented, the pleasantness of that activity). Six goods were presented from each of the four categories randomly interleaved with an inter-trial interval (ITI) of 6, 8, or 10 seconds (s) drawn randomly with uniform probability. Similar to the viewing/valuation paradigm used by Levy et al. [7], on 12 random “question trials” (one of the 12 presentations for each of the non-environmental and non-activity goods), after the 2 s fixation, subjects were asked whether they preferred the good they had just seen or a random amount of money (ranging from $1 to $10). The response had to be made within 1.5 s, and was followed for 0.5 s by feedback—either the name of the good or “money,” depending on their selection. If the subject did not respond within the 1.5 s, the feedback “no response” was presented for 0.5 s (Fig 1b). Subjects were told that one of these question trials would be randomly chosen at the end and that they would receive their selection on that trial—the good or the money. Because these trials were only included to maintain subjects’ concentration on the valuation task, they were excluded from all subsequent analyses. In addition, environmental proposals and daily activities were never presented as question trials, to avoid potential confusion.

As a replication of the previous studies that involved these stimuli, snack foods and daily activities were presented as text [12], and consumer goods were represented by pictures of the goods accompanied by text [7]. Before entering the scanner, subjects were presented with the snack foods and consumer goods briefly to ensure they were familiar with the items. Similarly, for the environmental goods, subjects were verbally briefed outside the scanner on the environmental issues at hand (Fig 1a, but see S1 File for full proposals) and asked to consider their willingness-to-pay for each proposal following it’s presentation. While subjects were inside the scanner, environmental proposals were presented again, represented by the visual aids that accompanied each proposal during the initial verbal presentation. While subjects viewed these goods, BOLD activity was measured using fMRI (Fig 1b and 1c).

**Fig 2:**
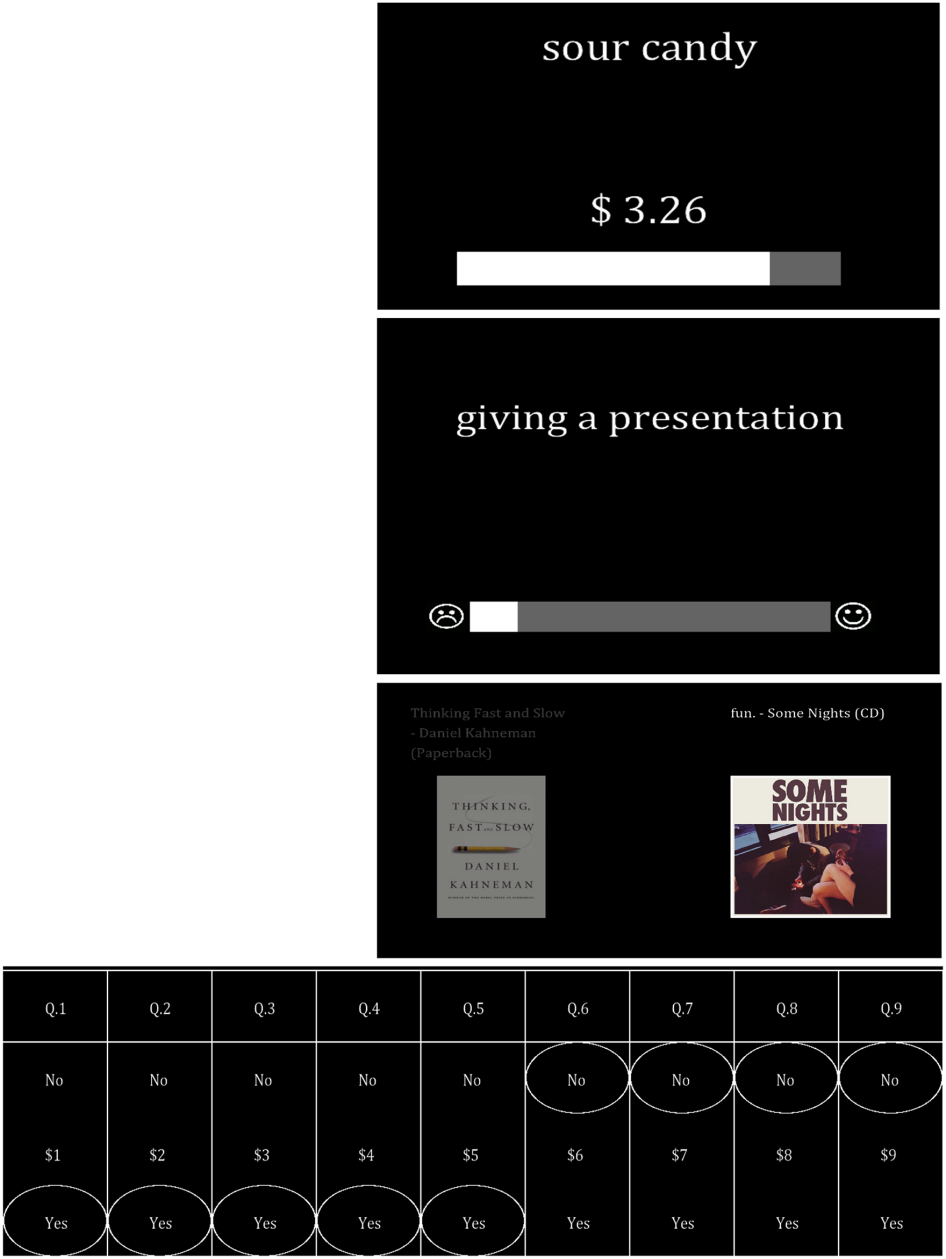
Example screens of behavioral value-elicitation procedures. From top to bottom: auction bidding with snack foods, visual analog scale with daily activities, choices between consumer goods, and payment card response screen for environmental proposals.

In the second stage of our experiment, subjects were taken out of the scanner and asked to place values on all four categories of goods using four different valuation procedures (Fig 2):

1. Snack foods were valued using the incentive-compatible Becker, DeGroot & Marschak (BDM) auction [24–25]. BDM auctions elicit an individual’s maximum willingness-to-pay with rules that incentivize truthful responses [26]. After all the valuation procedures were complete, one trial was randomly selected and implemented; in the event the subject’s bid was higher than the randomly selected price, the subject purchased the good at the random price.
2. Consumer goods were valued using an incentive-compatible choice experiment [7]. In this task, individuals simply chose which item they wanted more from all pairwise combinations of our consumer goods. After all valuation procedures were complete, one trial was then selected randomly, and the subjects received the good they chose on that randomly selected trial.
3. Daily activities were rated for pleasantness using a non-incentive-compatible Likert-type scale [12]. Here, subjects were simply asked to indicate how pleasurable a particular activity would be on a unitless slider bar scale. Identical to Gross et al., 2014, the slider bar was accompanied by a smiling and frowning face symbol on each end to indicate the valence of possible judgments.
4. Environmental proposals were valued using the CV procedure utilizing a standard payment card format [27]. The payment vehicle was described as an increase in the utility bill of taxpayers in the event that these hypothetical policies are enacted. Subjects were asked to respond “yes” or “no” to varying amounts of potential costs associated with each proposal.

All trials belonging to each preference-elicitation procedure were randomly interleaved during the behavioral session, similar to the presentation of each good type during the scanning session. Trial order for goods was randomized across subjects.

## Results

### Trial-related neural activity during viewing of goods and activities

**Fig 3:**
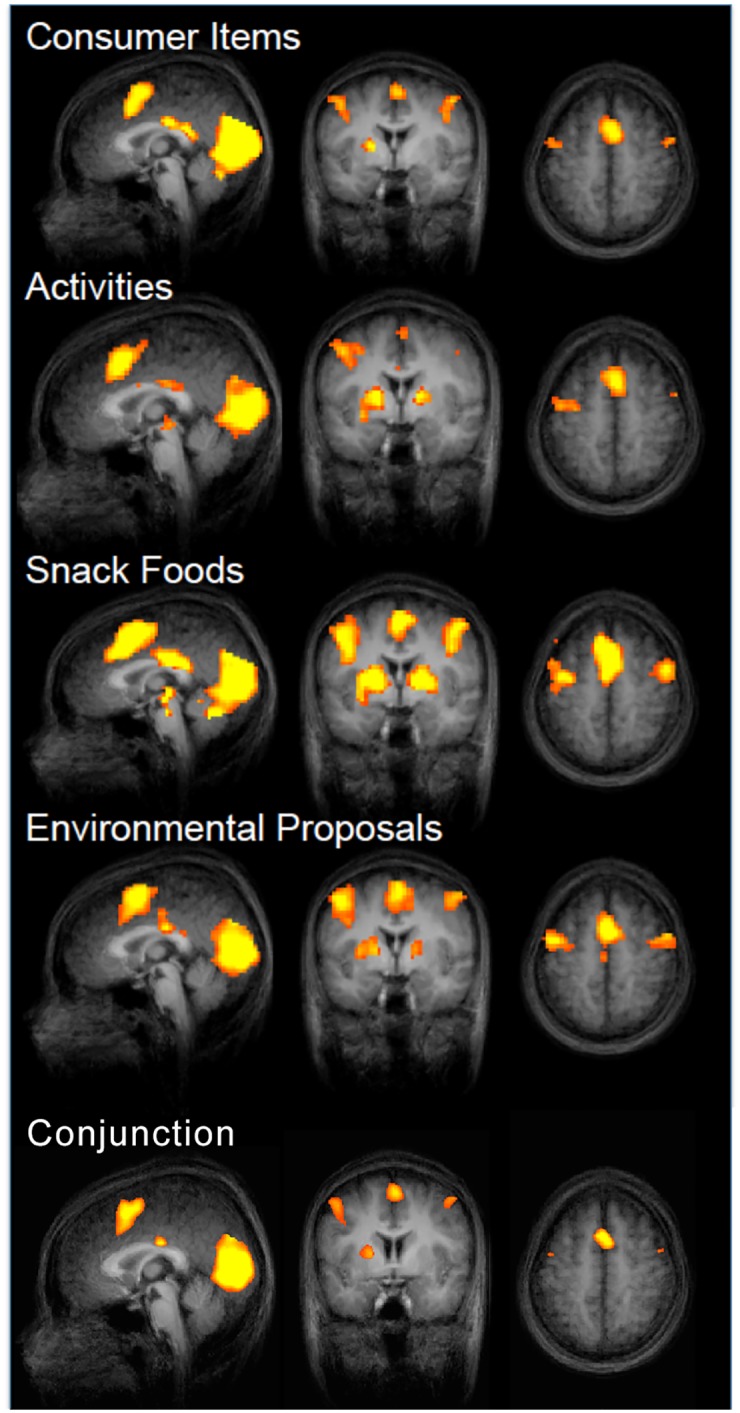
A nonparametric regression for each trial type in contrast to inter-trial intervals reveals identical activations (p < 0.001, FDR corrected) within dorsomedial prefrontal cortex (dmPFC), posterior cingulate cortex (PCC), ventral striatum, visual cortical areas, and superior frontal sulcus.

First, we examined the trial-related activity belonging to the *presentation* of our four separate classes of “goods” (snack foods, consumer goods, daily activities, and environmental proposals). This allowed us to visualize the average pattern of BOLD activation under each of these four viewing conditions. As shown in Fig 3, a univariate regression analysis using the trial onsets of each trial type (snack foods, consumer goods, daily activities, and environmental proposals) as separate regressors revealed robust and statistically indistinguishable activations of a number of areas: the dorsomedial prefrontal cortex (dmPFC), posterior cingulate cortex (PCC), VS, visual cortical areas, and superior frontal sulcus. A conjunction analysis confirms the activation of these complete circuits using the contrasts belonging to each trial type (snack foods ∩ consumer goods ∩ daily activities ∩ environmental proposals, p < 0.05, FDR corrected). These results replicate earlier studies in terms of the overall activation pattern elicited by snack foods, consumer goods, and daily activities [7,12,26]. We show here for the first time that environmental proposals yield a very similar pattern of overall brain activation during the trials. Further, this pattern of activation observed for all four goods is similar to previously established demonstrations of the executive attentional network [28]. Though we do not observe the activation of the posterior parietal region (a region associated traditionally with attention) in this analysis, a value-based parametric regression suggests that this region might be predominantly modulated by value during the current task, rather than attention/task engagement alone.

### Behavioral Valuation Trials

**Fig 4:**
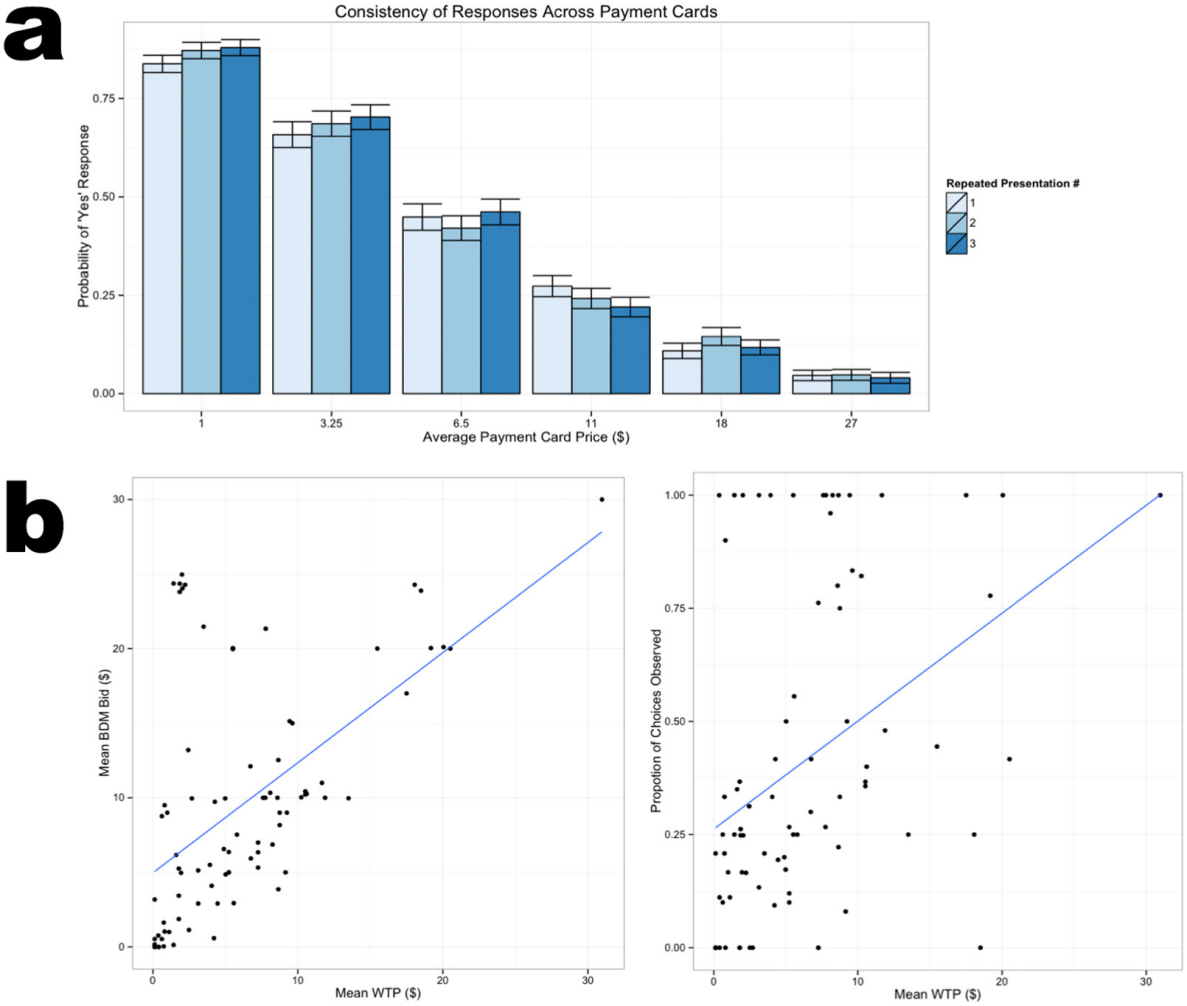
(a) Choice probabilities of subjects for each payment card range did not vary significantly across any and all repetitions that were randomly interleaved across the behavioral session. (b) Correlations between alternative measures of environmental preferences. An extended sample (N = 15) submitted bids and choices in addition to the payment card with the exact same environmental proposals as a measure of consistency. Left: Correlation between mean WTP willingness-to-pay and mean BDM bid for each proposal (*r* = 0.55, p < 0.01). Right: Correlation between mean willingness-to-pay for each proposal and choices observed (*r* = 0.39, p < 0.01) between environmental proposals.

Our preference-elicitation measures yielded four different behavioral response types from which we could aggregate and associate with the neural data. To assess the reliability (i.e., consistency of subjects making the same response across repeated measurements) with which subjects reported the values for the CV payment cards, we computed the probabilities of a “yes” response across each of the repeated presentations of each of the payment cards during the behavioral valuation session (subjects were presented with payment cards with different ranges of cost interleaved with all other methods, repeated three times for each cost interval). Fig 4a shows that the probability of a “yes” response did not change significantly across any and all repetitions of the same payment card. The validity of the payment card responses was additionally supported by data from a subset of individuals (N = 15) who responded with choices and bids toward identical proposals after the experiment. In this investigation, subjects performed an additional final behavioral valuation task that involved choosing between, and bidding on, the same environmental proposals (after having completed the payment cards for the same proposals and other preference measures for their respective goods). The resulting responses were highly correlated irrespective of the response format of the environmental survey (Fig 4b). Since the other three behavioral methods have been widely studied by neuroscientists, we did not attempt to establish their reliability in the present study.

Returning to our main experiment, the “yes” responses obtained from each payment card were used to compute a willingness-to-pay for each environmental proposal for each subject. The remaining preference procedures provided us with an ordered set of idiosyncratic preferences for each subject across each of the other three good types. This was achieved by computing the average auction bids for snack foods, computing the average pleasantness ratings for each activity, and by tallying the number of choices made toward each good (since all goods were compared against each other, the total number of times each good was chosen serves as a nonparametric indicator of the ordinal rank of each good in the choice set). All four of our preference-elicitation measures successfully elicited a reliable set of idiosyncratic preferences within each subject across each category of goods.

### Parametric regression relating behaviorally measured preferences to neural activity

**Fig 5:**
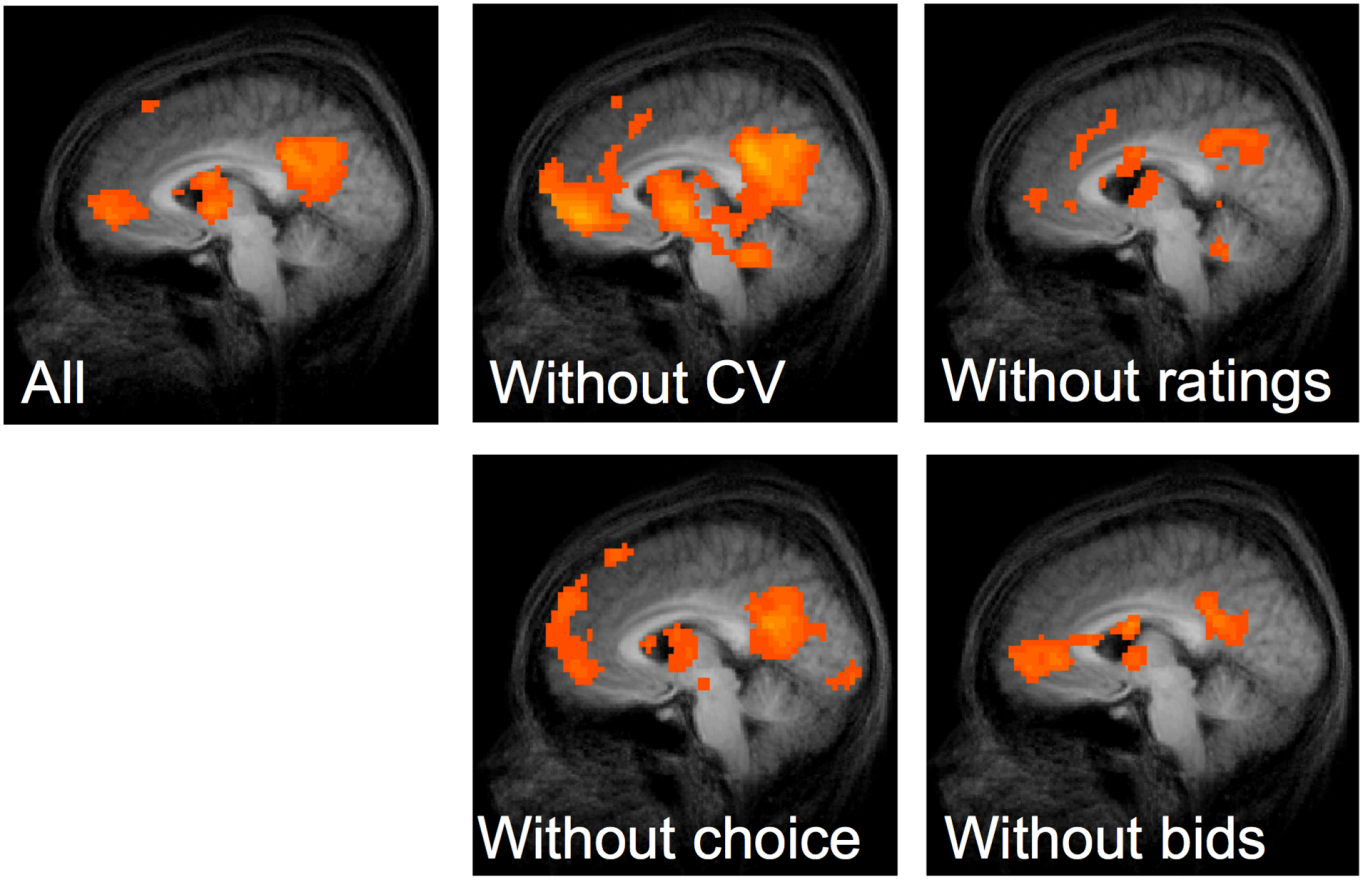
Statistical parameter maps for parametric regression including all behaviorally measured preferences as regressors and all possible “leave-one-out” combinations, replicating neural correlates identified in previous studies and meta-studies of subjective valuations. Clockwise from top left: Inclusive of all preferences, leaving out contingent valuations, leaving out activity ratings, leaving out snack food bids, and leaving out consumer good choices. Threshold for each map was set to p < 0.01, FDR corrected.

The set of behavioral valuations for *all four categories of goods* (using all four techniques) were then used to generate parametric estimates of *subjective value* (specific to each individual subject) to correlate with *all* brain activity measured during viewing of all of the goods. A whole-brain univariate regression analysis reveals that BOLD activity measured across all four categories of goods correlates with preference rankings in regions of the VS, ventromedial prefrontal cortex (vmPFC), and posterior parietal cortex (PPC)—all areas that have been implicated in subjective valuation meta-analyses [22–23] as well as in the original studies that we replicated within this exercise [7,12,26]. This correlation is significant (p < 0.01, FDR corrected) when inclusive of all the parametric regressors, as well as across a leave-one-out procedure excluding any individual set (i.e., any individual type of good) of preference regressors (Fig 5). Our overall neural correlate of subjective valuation thus replicates previous decision-making fMRI studies involving different types of rewards and valuation [19,22,23].

**Fig 6:**
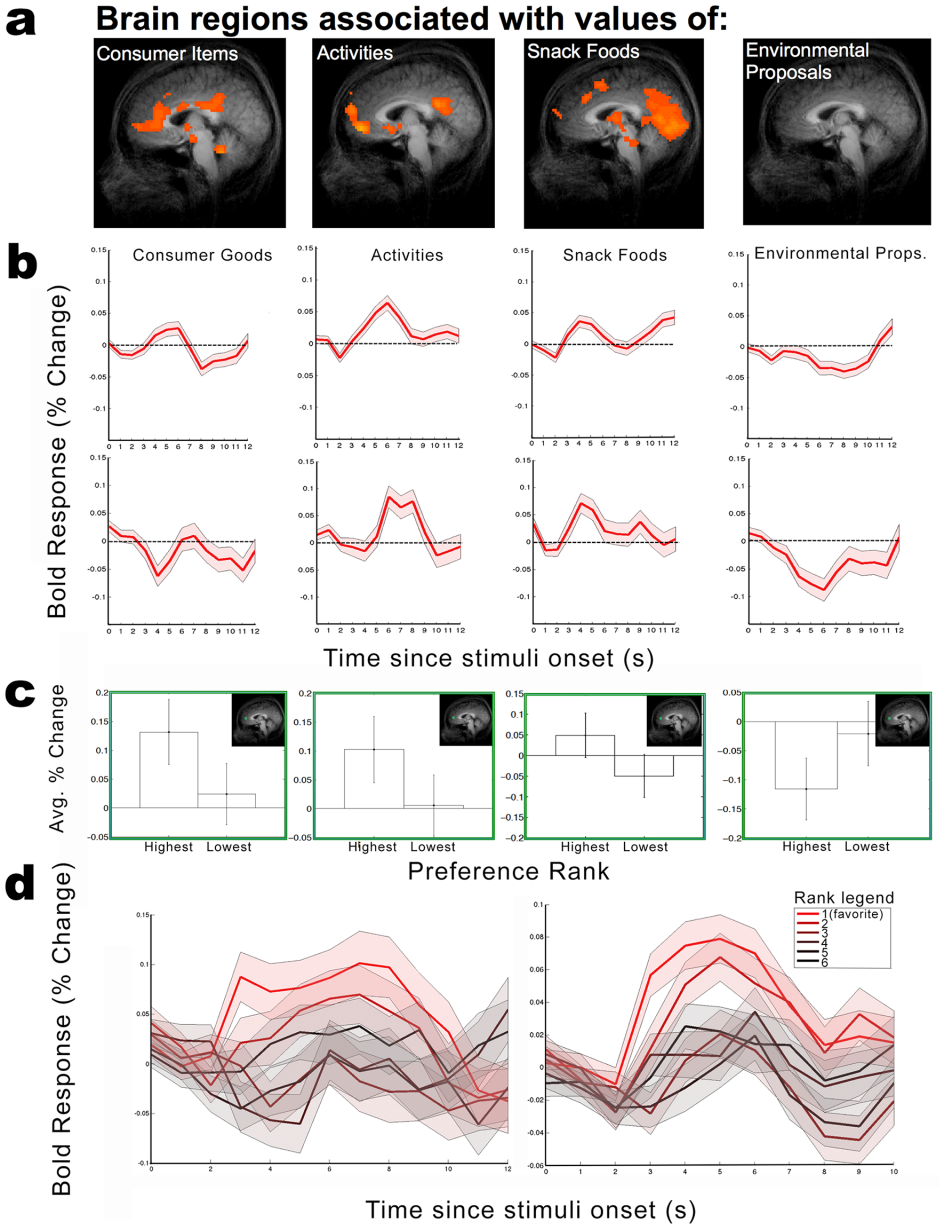
(a) Statistical parameter maps revealing regions associated with subjective value of different categories of goods (p < 0.05, uncorrected). (b) Average time course following the onset of each trial type for spherical ROIs centered on the ventral striatum (top) and vmPFC (bottom). (c) Average time course averaged according to good rank from the vmPFC (left) and ventral striatum (right). (d) Differential activity within vmPFC region of interest (inset) for highest and lowest ranked goods (error bars indicates ± s.e. of the mean). Inset: spherical vmPFC ROI used to compute average change BOLD activity between time points 5–8 after stimuli onset.

Next, in order to assess the contribution of *each class* of valuation procedure and object type to this overall correlation, and to allow comparison of the subjective value representations across good types, separate contrast maps (Fig 6a) were generated to relate the behaviorally derived valuations from each individual method to the neural activity elicited by their respective good types during their viewing in the scanner. Here, once again, significant correlations were observed (either vmPFC, VS, or both) for valuations of snack foods, consumer goods, and daily activities (p < 0.05, uncorrected), replicating their respective fMRI valuation studies. Surprisingly, however, we observed no statistically significant correlation between the BOLD activity associated with viewing environmental proposals and elicited environmental preferences.

### BOLD Time-course Visualization

In order to visualize the measured BOLD time courses following the onset of each trial type, we computed the percent BOLD change from an average baseline window of 2 TRs (4 s) preceding each trial. In comparing the brain’s response within each trial type, we utilized an a priori—defined region of interest (ROI) for the vmPFC (see below), and a 3 mm sphere centered at the peak T-statistic of the value correlating region within the ventral striatum (Talairach coordinates X = 9, Y = −4 and Z = −2). The time courses for each trial type reveal a reduced level of activity for environmental trials for both of the investigated regions (Fig 6b).

### Independent ROI analysis

Here, we focus our analysis on the vmPFC as a hypothesized region for common valuations (e.g., Levy & Glimcher, 2012). In order to examine a further property of valuation-associated BOLD signals, we tested for differential activity associated with high- and low-valued goods. Here, we used a spherical ROI with a 3 mm radius from the vmPFC decision contrast (Bartra et al., 2013 [22], with Talairach coordinates centered at X = 2, Y = 36, Z = −8). Replicating the protocol of measuring percent BOLD change as a proxy for subjective value [7], we computed the average percent change in BOLD for the most and least preferred goods within their respective categories using time points 3, 4, and 5 after stimulus onset (i.e., 6–10 s after stimulus onset). The resulting averages show differential BOLD activity associated with behavioral valuations of snack foods, consumer goods, and daily activities. Again, we found no evidence for average differential neural activity within valuation areas for the CV-elicited behavioral valuations (Fig 6c). We also computed the average time courses belonging to each good according to its rank (Fig 6d), to observe the actual differences in activation between goods that were (subsequently) behaviorally declared to be high or low in value.

**Fig 7:**
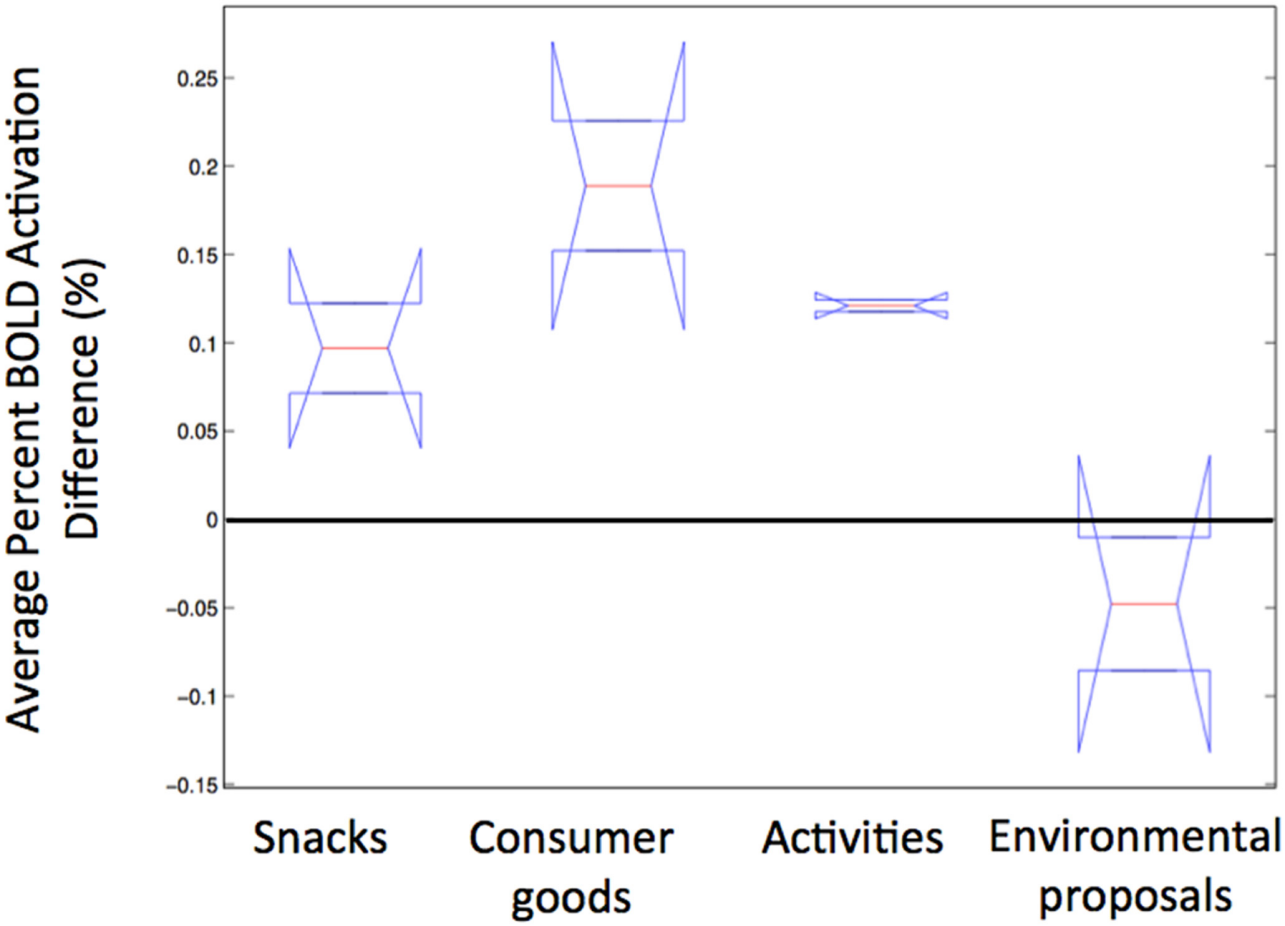
Boxplot of average differences in activation between most and least preferred goods across all four categories of goods. Group averages were computed from differences in vmPFC BOLD activation between most and least preferred goods (i.e., differences in average percent BOLD change within time points 3, 4, and 5 or, alternatively, 6–10 s after stimulus onset) for each subject within each good category. A one-way ANOVA performed across the groups suggests that the between-group means to be significantly different (F = 11.58, p < 0.05). Excluding the environmental proposals eliminates this effect (F = 3.41, p > 0.1), suggesting that the remaining three group means are not significantly different from each other.

We used two separate one-way ANOVAs to quantify this difference observed in the environmental goods category. To do this, we computed the average difference in vmPFC BOLD activation between most and least preferred goods (i.e., differences in average percent BOLD activation within time points 3, 4, and 5 or, alternatively, 6–10 s after stimulus onset), for each subject within each good category (the population averages of which are plotted in Fig 6c). A one-way ANOVA performed on the differences in activation observed with data from all four categories (Fig 7) suggests that there is a significant difference in the groups (F = 11.58, p < 0.05). A second ANOVA performed without the environmental activations suggests that there is no significant difference in variation within the remaining three groups of preferences (F = 3.41, p > 0.1). Thus, CV-based estimates of environmental preferences differ in their brain-behavior association from the three other procedures and categories of goods we examined.

To further explore the relationship between neural activation and CV-based valuation, we compared the auction bids for snack foods with CV-elicited willingness-to-pay for environmental goods. Put simply, we examined how much more the subjects were willing to pay for the environmental goods than for the snack foods, and how this difference in monetary valuation related to differences in brain activity within the a priori—defined vmPFC ROI.

**Fig 8:**
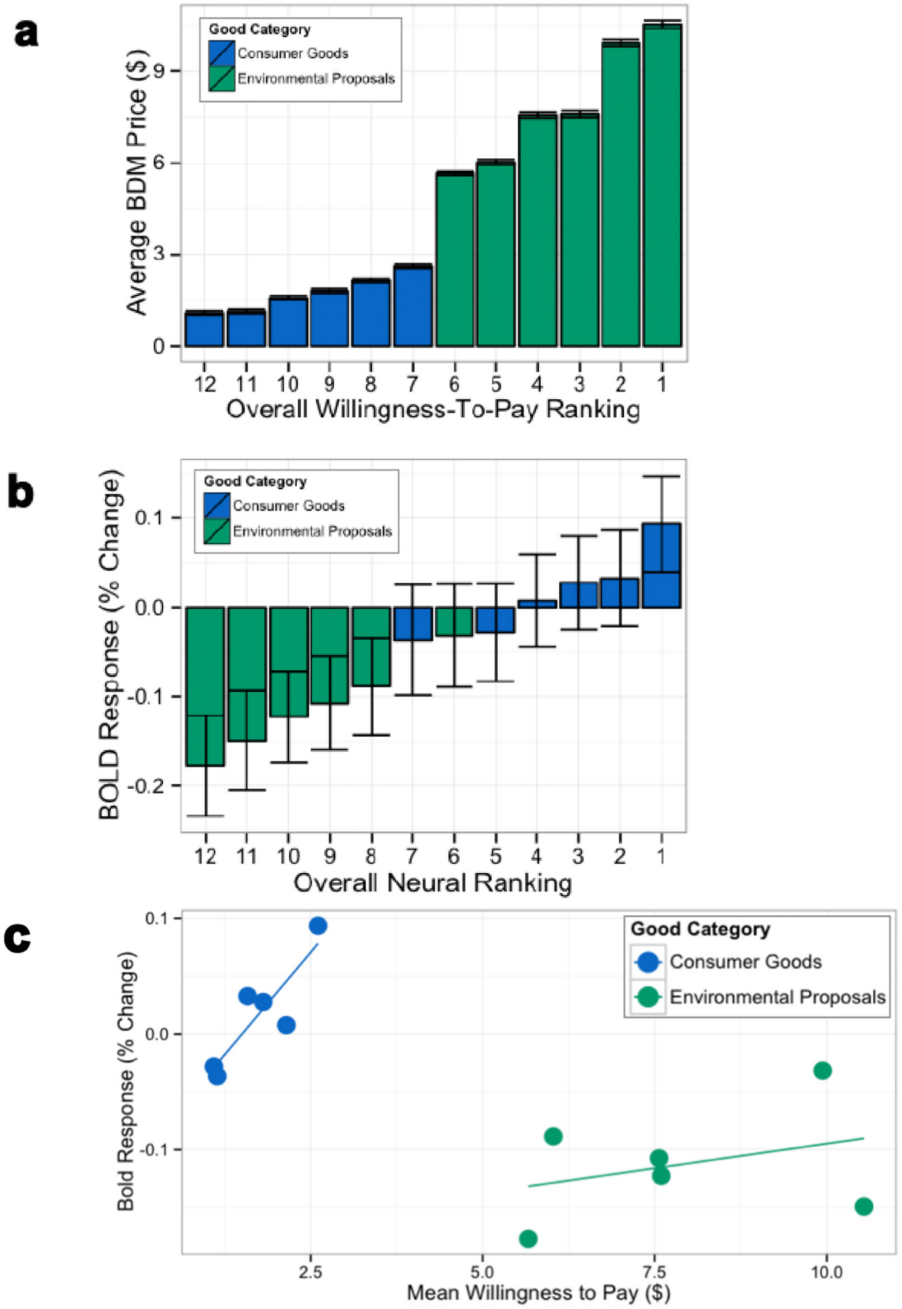
(a) Average willingness-to-pay computed from behavioral responses in BDM auction for consumer goods and payment card responses for environmental proposals (error bars indicates ± s.e. of the mean). (b) Average change in percent BOLD response following the presentation of consumer goods or environmental proposals. (c) The correlation for BDM-consumer goods pairs is significant (r = 0.88, p < 0.05), in contrast to the neural correlation for CV of the environmental proposals (r = 0.34, p = 0.51).

Fig 8a shows, as a population, how much subjects report valuing each of these goods—with the environmental goods receiving much higher valuations on average. Based on previous findings that an increase in the BOLD signal within the vmPFC is correlated with increases in preferred-ness (Levy et al., 2011), we analyzed the peak activations from the vmPFC when subjects viewed both classes of goods. Within the 6–10 s interval that was analyzed earlier, the time point of 8 s after stimuli onset yielded the greatest percent BOLD signal increase (on average) across both categories. We rank-ordered these average changes in BOLD signal in Fig 8b. Note that environmental goods yield consistently lower mean activations than snack foods despite the greater monetary values placed on these goods during the CV procedure. In Fig 8c, we compare these averaged changes in signal and valuations directly; a positive and significant correlation was observed between value and activation for snack foods in this canonical valuation ROI, but a markedly weaker relationship obtained for CV-evaluated environmental public goods.

In sum, there exists for all previously studied goods a stable behavioral valuation, and a relationship between that valuation and neural activity in classical value areas. However, the average activity in these areas elicited by the environmental proposals is lower and ordered differently from what might have been inferred from the behavioral measurements elicited by the CV procedure.

### Sub-population analysis of environmental preferences

**Fig 9:**
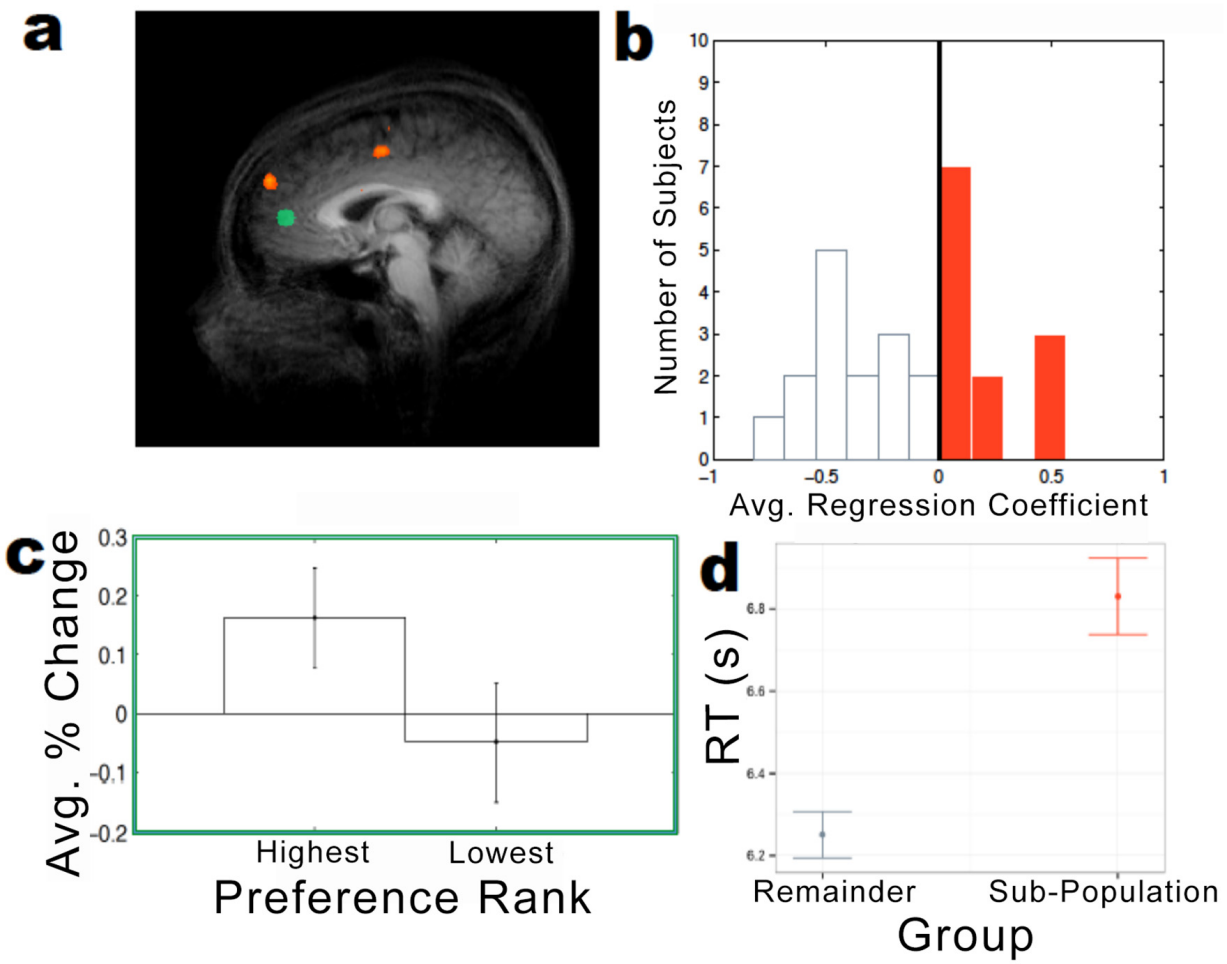
(a) Regions correlated with environmental preferences (p < 0.05, uncorrected) for exploratory sub-population (orange) relative to a priori vmPFC region of interest (green). (b) Selection of exploratory sub-population based on average whole-brain regression coefficient for environmental proposals. (c) Differential activity within exploratory sub-population’s vmPFC region for favored vs. nonfavored proposals. (d) Reaction time differences between exploratory sub-population and remaining subjects.

To investigate more deeply the relationship between behavioral valuations elicited by the CV procedure and neural activity, we performed a within-subject regression on CV-elicited valuations and selected all subjects who showed a positive (though not necessarily significant) correlation *globally* (averaged across all voxels) for each whole brain (Fig 9b). Once these individuals were identified (N = 12), the same random-effects regression that was performed previously on the whole sample was re-run within this sub-population to test for statistically significant correlations. Using this admittedly unconventional procedure, we were able to identify a sub-population of subjects that exhibited a positive correlation (p < 0.05, uncorrected; though one must be cautious in interpreting this statistical significance given the post-hoc selection process) in a single region of the brain (Fig 9a). Surprisingly, this correlation between environmental valuations and brain activity was observed not in a valuation-associated area but rather in the *dorso*medial prefrontal cortex (dmPFC). Indeed, using the same percent change analysis employed earlier, the vmPFC signal for this sub-population did not yield significantly differential activity between most and least-preferred environmental goods (Fig 9c). This finding suggests that there are, as expected, brain activations associated with the CV-elicited preferences, but it strengthens the conclusion that these correlations do not lie within traditional valuation areas. It should also be noted that at a post-hoc behavioral level, this group exhibits longer CV-trial completion times ($\overset{-}{x}$ = 6.83, s.d. = 0.09) compared to the remainder of our sample ($\overset{-}{x}$ = 6.23, s.d. = 0.06, Fig 9d). Subsequent to the completion of this analysis, a median split of the subjects using the response time (RT) distribution (selecting for the slower half of subjects, with average completion times greater than *Md* = 6.55 s) also yielded a similarly significant correlation between environmental valuations and activity in the dmPFC (p < 0.05, uncorrected) for this sub-population. It is important to note, however, that this supplementary analysis was performed as a subsequent validation exercise and thus should not be interpreted as a second piece of independent evidence for a sub-population effect.

## Discussion

We measured the neural activity associated with a range of stimuli that included environmental goods, snack foods, daily activities, and consumer goods and examined how this activity related to series of behaviorally elicited valuations of these goods. Firstly, we found that the presentation of each class of goods produced identical activations throughout what has come to be known as the brain’s executive *attentional network* [28]. Indeed, the overall patterns of activity that we observed (time-locked to stimulus presentation) were statistically indistinguishable for all four categories of goods. This is, to our knowledge, the first time that environmental goods have been examined in this way.

We also examined the behavioral valuations that subjects placed on these four classes of goods using four very different procedures. We examined the relationship between neural activity and the four different types of behavioral valuations we elicited. We examined this relationship in two ways: using a whole-brain analysis that searched for any correlation between preferences and the BOLD signal, and using an ROI-based approach that targeted valuation-associated areas. Both approaches revealed a previously documented correlation between brain activity in valuation areas and behaviorally elicited valuations for three classes of goods. These were the relationships between: i) snack foods and auction bids, ii) daily activities and pleasantness ratings, and iii) consumer goods and choice-based rankings. Surprisingly, we saw no significant correlations between activity in any valuation-associated area and the CV-elicited willingness-to-pay valuations environmental goods. We did, however, observe a limited correlation between the CV-elicited valuations and activity in the dmPFC (specific to a subset of subjects). However, this particular region has not been associated with subjective valuation in decision making; instead, it has typically been associated with the deployment of cognitive control, task switching, and shifting decision strategies.

We take these results to indicate that the brain activity associated with the elicitation of environmental values by the CV procedure differs profoundly from the brain activity associated with all other known good-valuation relationships. Below, we highlight several possible explanations for this unexpected result. Broadly speaking, these possible explanations highlight: i) the distinctive nature of environmental proposals relative to other previously studied classes of goods, ii) possible heterogeneity across subjects in valuing the environmental proposals, iii) a substantial limitation in contemporary neuroimaging technology, and iv) potential limitations in the current experimental design.

### Environmental public goods possess inherently different properties than all previously studied good categories

Without a doubt, environmental public goods possess distinct properties that make them difficult to appraise behaviorally using traditional methods. Previous studies of neural and behavioral valuation in experimental public goods games [15] as well as research on contributions to charities that produce public goods [14] suggest that fMRI activation patterns for these public goods are similar to those for private goods. Thus, the valuation of public goods per se can activate traditional value areas. However, to the best of our knowledge, previous fMRI studies on public goods have not examined the neural representation of large-scale environmental proposals or categories of goods with considerable passive-use value. Goods of this type may invoke separate neural processes from those that have been observed in previous studies of decision-making and valuation.

### Subjects may employ a variety of neural valuation strategies when engaged in the behaviorally defined CV procedure

Our failure to observe a clear correlation between the CV-elicited valuations for environmental goods and activity in valuation areas may be caused by a between-subject heterogeneity in strategies. It may be that different subjects employ different neural mechanisms for contingent valuation, and as a result, the aggregated data from a group of heterogeneous subjects lacks the statistical power to reveal these disparate valuation mechanisms. The sub-population (N = 12) isolated from our study group that exhibits correlations within the dmPFC (Fig 8) with CV-elicited valuations may argue for this interpretation. Curiously, however, dmPFC is not traditionally associated with valuation but rather with “shifting of control” among “decision strategies” [29] as well as with “cognitive control” more generally [30]. The slower behavioral reaction times (during payment card trials) exhibited by subjects showing this effect may support this conjecture.

### Contemporary scanning technology is unable to image activity related to the subjective valuation of environmental public goods

Another possibility is that the contingent valuation of environmental public goods does, in fact, correlate with activity in certain brain areas (indeed, at some level of analysis they must). However, the statistical power needed to detect this correlation might be far greater than that required for the other goods included in this study. Value signals for environmental goods may have been normalized during the experiment within a larger set of familiar private goods such that the value-related activations of the environmental goods were rendered undetectable by fMRI. Such contrast issues would, however, also suggest that environmental public goods are valued on a vastly different internal scale than what is elicited by traditional preference experiments. Our observation in Fig 8c that the relationship between neural activity and behavioral valuation for snack foods/money versus environmental goods/money is so profoundly different in the vmPFC may argue for this interpretation. Related to this argument, the hypothetical nature of the proposals may have lowered the magnitude of measured value signals, as has been observed in previous fMRI valuation experiments involving hypothetical and actualized outcomes [31].

### Limitations in the current experimental design with respect to environmental valuations

Our efforts to study contingent valuation in a similar manner to the other valuation procedures impose several important limitations. It should be noted that making the trial durations, presentation formats, and other design parameters similar to other procedures used for other classes of goods may have inadvertently obscured a correlation between the CV derived environmental values and the brain activations we measured. Indeed, adjustments to the briefing procedure and presentation styles of CV visual aids have been studied with respect to CV’s validity [32]. Likewise, the current study offers a starting point for future examinations of neural activity and environmental valuations. It may be that value signals in the brain for environmental goods may be measurable on a different temporal or spatial scale than all other economic goods studied previously. Thus, our study cannot offer anything like a complete theory of this class of valuations and goods. At the very least, these results call for future studies employing novel experimental designs calibrated solely toward the valuation of environmental goods.

## Summary

Our results indicate that no particular brain region exhibits significantly measurable correlated activity with the subjective values estimated via contingent valuation of environmental proposals using our procedures. This observation lies in stark contrast to all other known goods/valuation procedures for which such correlations have already been observed using these same approaches. This was true despite the fact that we briefed subjects on the environmental proposals, that the neural data indicates that our subjects were attentively viewing the stimuli, and that the behavioral survey elicited reliable responses. The absence of such a correlation is thus puzzling, especially given that this observation was made while successfully replicating, on randomly interleaved trials, three disparate classic valuation results [7,12,26]. Nevertheless, the environmental preferences we obtained do not correlate with brain activations observed in valuation circuits during the viewing of environmental goods. This finding strongly suggests that the domain of CV for environmental goods presents a clear uniqueness in relationship of behavioral valuation to brain activity.

This result may be of interest because CV is frequently used for informing public policy and litigation. External validity studies, where available, do suggest that the CV method is demand-revealing when conditions related to incentive compatibility are met [33]. The current results do not challenge the established validity of the methodology. However, the anomaly we have revealed—the finding that, at a neural level, CV for environmental valuation is very different from all other known valuation methods/goods pairings—does call for more work at the intersection of contingent valuation and neuroscience, in tandem with the ongoing efforts of environmental economists to further pinpoint factors behind effective implementation of contingent valuation.

## Supporting Information

S1 Fig Environmental Proposals. All six environmental proposals and their proposed payment vehicles that were presented to subjects before the fMRI scanning sessions and during the behavioral valuation task.
(PDF)

S1 File Supplementary Methods. (DOCX)
