# Supplementary material for: The Measurement of Subjective Value and Its Relation to Contingent Valuation and Environmental Public Goods
Source: PLoS One. 2015 Jul 29;10(7):e0132842. doi: 10.1371/journal.pone.0132842 (PMC4519262; doi:10.1371/journal.pone.0132842)
Supplement: S1 Fig — All six environmental proposals and their proposed payment vehicles that were presented to subjects before the fMRI scanning sessions and during the behavioral valuation task. (PDF) [file pone.0132842.s001.pdf]

Sample Instructions

We thank you for agreeing to participate in this study. As compensation for your time and effort, you will receive $40. This amount will be paid privately to you, in cash, at the end of today's session. Research foundations have provided the funds for this study.

You will never be asked to reveal your identity to anyone during the course of the study. Your name will never be associated with any of your decisions. In this study we are asking you to complete a survey.

You are one of a small number of people who are being asked to share their preferences and views about local policy issues. In particular, you will be asked how much of a fee you would be willing to pay on your monthly utility bill, for the foreseeable future, to support certain environmental programs.

Note that no money will be collected or payments made as a direct result of the decisions you will make.

However, the results of the research will be shared with the relevant policy makers. It is therefore possible that your decisions will be used to inform policy, and in this sense you may incur costs in the future. We ask that you make every effort to respond exactly as you would if, for instance, you were voting on related public referenda (ballot measures).

You will now be asked to make decisions on six proposals. Before you respond, we ask that you read the presented information very carefully as you may not be familiar with the specific issues at hand.

These proposals are not alternatives to one another. The chance one proposal is actually implemented does not depend on whether the others are implemented. Therefore, we ask that you please consider each proposal in isolation, and do not make decisions on one proposal based on the decisions you made on the others.

Proposal 1: GREEN POWER IN NEW YORK

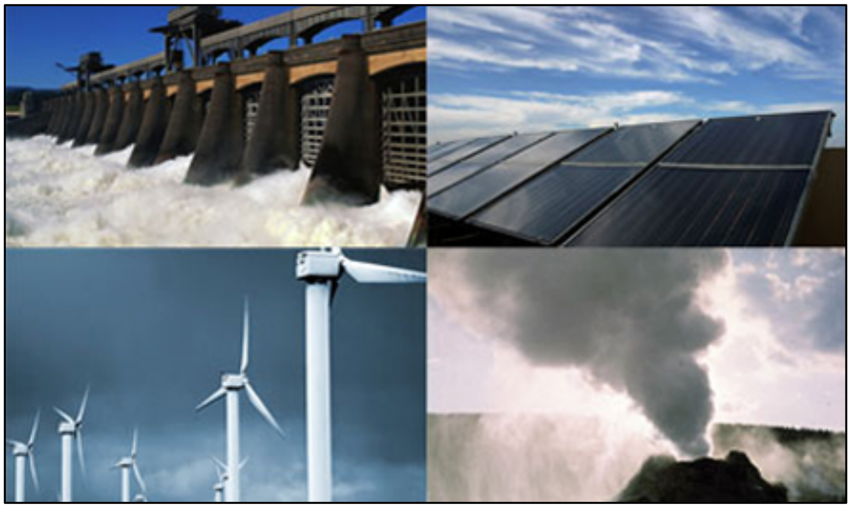

New York state uses a variety of energy sources to meet its demand for electricity. "Green Power" is the portion of electricity that is produced using renewable energy sources including (but not limited to) hydropower, wind, solar, and geothermal energy. Collectively, these sources comprise about 30% of total energy use in the state.

As renewable energy, Green Power produces fewer air pollutants and greenhouse gases than fossil fuel energy. The use of green power sources also helps to diversify the fuel supply, increasing the reliability of the New York State electric power system and contributing to more stable energy prices.

Currently, both the state government and electrical utilities are devoting funds to increase the portion of New York's electricity that comes from Green Power. Among other efforts, these funds support research into improving renewable energy technologies, along with the construction and continued maintenance of Green Power generation facilities and associated infrastructure.

Further funding is needed to support the development of Green Power. We would like you to consider the following proposal that would, if implemented, lead to an increased portion of New York state's electricity coming from Green Power:

As part of New York's strategy for the continued expansion of Green Power sources, local utility companies might implement a program to which customers would financially contribute.

The objective of such financial support would be to increase New York's total Green Power percentage from 30% to 40%

If the proposal were to pass, funding would come from a monthly, itemized addition to the utility bill of all New York ratepayers (including those who voted against the proposal). Suppose that the money collected will be managed honestly and allocated in full to increasing the state's Green Power supply.

The cost of implementing this policy is at present not certain. Because of this, we are asking you about several possible cost amounts. When the cost of implementing the proposal is known, the policymaker will be able to look at the votes associated with the actual cost and use this information to help make a policy decision.

What is your vote on the proposal, given that passage of the proposal would cost you these amounts per month? *(Please indicate a Y or N response for each amount by clicking on the desired response).*

Proposal 2: RESTORATION OF THE BRONX RIVER WATERSHED

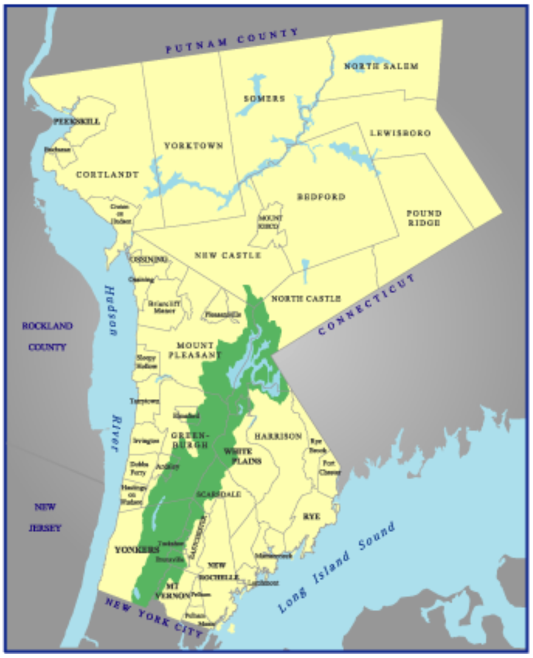

A watershed is the area of land from which the surface water runoff drains into the same body of water. The Bronx River Watershed extends from Kensico Dam in Westchester County to the East River and drains about 56 square miles.

Stormwater runoff from the urban watershed threatens the quantity and quality of the remaining wetlands in the Bronx. This runoff can cause erosion in the wetlands and prevent re-growth of native wetland plants. Additionally, the sediment in runoff can smother animal habitat and native vegetation. The pollution in urban runoff can also impact the wetlands, limiting the diversity and abundance of vegetation, aquatic invertebrates, and fish. Stormwater runoff can carry pollutants downstream from a variety of sources, including streets, pet waste, gardening chemicals, car washing, and flushing of harmful fluids into storm drains.

In 2004, construction began on the Bronx River Forest Floodplain and River Channel Rehabilitation Project. This project, with continued funding, will help repair the damage to the watershed and enhance in-stream habitat for plants and animals.

Further funding is needed to support the Bronx River Forest Floodplain and River Channel Rehabilitation Project. We would like you to consider the following proposal that would, if implemented, lead to the continued restoration of the Bronx River Watershed:

As part of New York's strategy for the continued restoration of the Bronx River Watershed, local utility companies might implement a program to which customers would financially contribute.

The objective of such financial support would be to ensure the restoration of 2/3 of the wetlands currently surrounding the Bronx River.

If the proposal were to pass, funding would come from a monthly, itemized addition to the utility bill of all New York ratepayers (including those who voted against the proposal). Suppose that the money collected will be managed honestly and allocated in full to restoring the Bronx River Watershed.

The cost of implementing this policy is at present not certain. Because of this, we are asking you about several possible cost amounts. When the cost of implementing the proposal is known, the policymaker will be able to look at the votes associated with the actual cost and use this information to help make a policy decision.

What is your vote on the proposal, given that passage of the proposal would cost you these amounts per month? *(Please indicate a Y or N response for each amount by clicking on the desired response).*

Proposal 3: HYDRAULIC FRACTURING IN NEW YORK

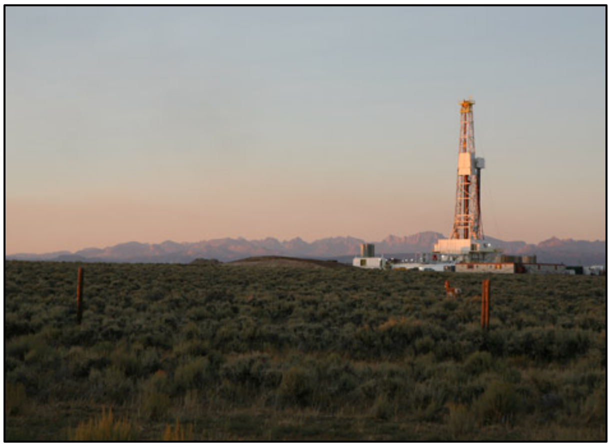

Fracking (short for hydraulic fracturing) is a drilling technique that involves injecting chemicals, sand, and water under high pressure directly into the ground to extract natural gas in shale deposits.

The injected mixture of chemicals and sediment, along with any natural gas released, may leak to the surface and flow into rivers and groundwater in the process or enter water supplies if the wastewater is disposed of in water treatment plants. Fracking can also release smog-forming and toxic air pollutants, as well as greenhouse gases, into the air.

Out of concern for the potential effects of fracking, New York state has commissioned a health impact assessment to aid in its decision about what sorts of regulations to impose on the fracking industry. The technique has just recently become common and the understanding of the full scope of its effects is still incomplete, but scientists are concerned about its potential effects on air and water quality.

New York has imposed a temporary moratorium on fracking but is considering allowing fracking to take place in the state.

Further funding is needed to support a program that the utility companies have implemented to allow ratepayers to pay an additional fee to avoid using natural gas extracted via fracking. We would like you to consider the following proposal that would, if implemented, lead to the replacement of fracking as an active drilling method:

As part of New York's strategy for the continued development and expansion of environmental assets, local utility companies might implement a program to which customers would financially contribute.

The utility companies would utilize the funding to develop energy sources other than fracking (either renewable energy sources or other, less invasive drilling techniques for natural gas). The funding would prevent 300 new fracking sites from opening in New York per year.

If the proposal were to pass, funding would come from a monthly, itemized addition to the utility bill of all New York ratepayers (including those who voted against the proposal). Suppose that the money collected will be managed honestly and allocated in full to decreasing the number of active fracking sites.

The cost of implementing this policy is at present not certain. Because of this we are asking you about several possible cost amounts. When the cost of implementing the proposal is known, the policymaker will be able to look at the votes associated with the actual cost and use this information to help make a policy decision.

What is your vote on the proposal, given that passage of the proposal would cost you these amounts per month? *(Please indicate a Y or N response for each amount by clicking on the desired response).*

Proposal 4: DEVELOPMENT OF A LARGE RESORT IN THE WESTERN CATSKILLS

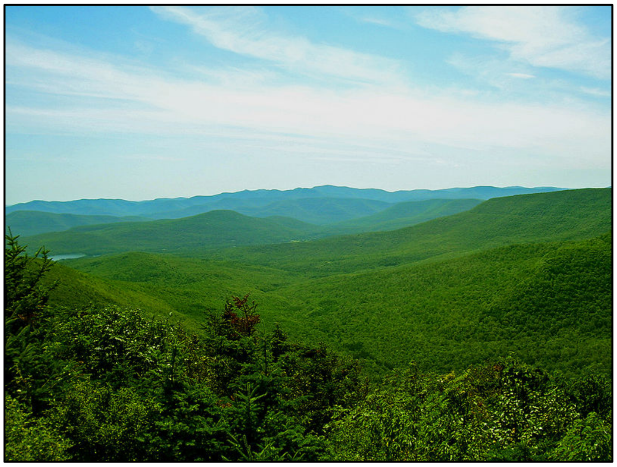

The Catskill Mountains are a large area in the southeastern portion of the state of New York. They are located approximately 100 miles north-northwest of New York City and forty miles southwest of Albany, starting just west of the Hudson River.

The region is rural, with little urban development, and contains rivers, forests, hiking trails, farmland, and habitat for many native plants and animals.

A developer proposes to build a large resort in the Western Catskills on a piece of land containing old-growth forest. Among other environmental benefits, this old-growth forest provides habitat for a number of native plant and animal species that are dependent on the old growth to survive, prevents soil erosion, and sequesters greenhouse gases out of the atmosphere.

Building the resort would eliminate this stand of old-growth forest and would also impact the environment through soil erosion during construction and increased traffic to the area after the resort and its new roads are open.

Further funding is needed to support the effort of environmental organizations to purchase a conservation easement on the land that would preserve the old-growth forest in the Western Catskills instead of selling the land to the resort developers. We would like you to consider the following proposal that would, if implemented, lead to the continued preservation of the current landscape structure of the Western Catskills:

As part of New York's strategy for the protection of rural land, local utility companies, in collaboration with local environmental organizations, might implement a program to which customers would financially contribute.

The utility companies would use this financial support to fund the purchase of a conservation easement that would stop the development of the proposed resort on the old-growth forest in the Western Catskills.

If the proposal were to pass, funding would come from a one-time, itemized addition to the utility bill of all New York ratepayers (including those who voted against the proposal). Suppose that the money collected will be managed honestly and allocated in full to preserving the landscape in the Western Catskills.

The cost of implementing this policy is at present not certain. Because of this we are asking you about several possible cost amounts. When the cost of implementing the proposal is known, the policymaker will be able to look at the votes associated with the actual cost and use this information to help make a policy decision.

What is your vote on the proposal, given that passage of the proposal would cost you these amounts [in a one-time payment?] ***(Please indicate a Y or N response for each amount by clicking on the desired response).***

Proposal 5: PROTECTION OF SEA TURTLES

Green Turtle

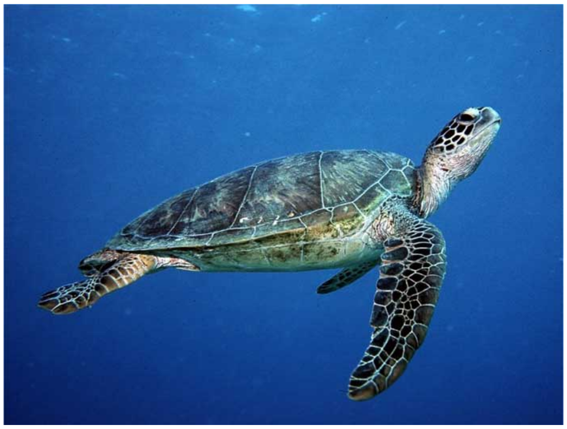

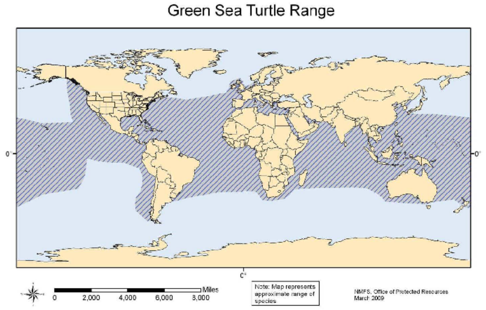

Olive Ridley Turtle

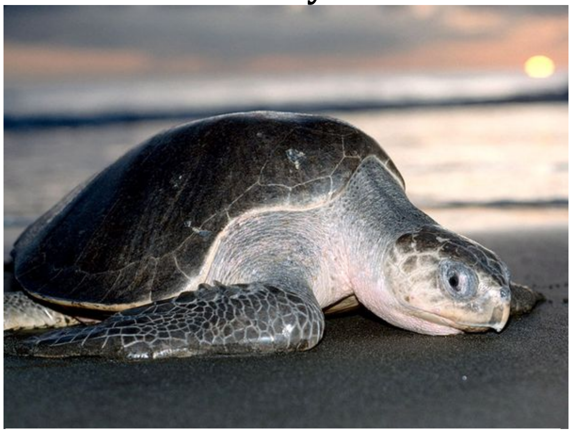

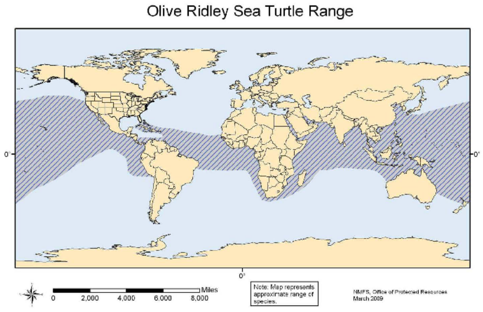

There are seven species of sea turtles worldwide, two of which are green turtles and olive ridley turtles. The populations of both species of turtles have declined substantially. In the past 100 to 150 years, the number of mature nesting green turtle females has declined by 48 to 65 percent, and the number of mature nesting olive ridley turtle females has declined by 50 percent since 1960.

The turtles are threatened by accidental capture in fishing gear, degradation of nesting beaches, disease, and human harvesting of eggs and turtles. Several policies have been implemented in the United States to protect sea turtles, including changes to fishing and shrimp harvesting practices to reduce sea turtle bycatch. Since 1989, the United States has banned shrimp imports from countries that do not protect sea turtles if turtles in those countries are at threat of extinction.

Sea turtles benefit from international treaties and agreements, including the Convention on International Trade in Endangered Species of Wild Flora and Fauna (CITES) and the Inter-American Convention for the Protection and Conservation of Sea Turtles (IAC).

Further financial support is needed for the enforced protection of sea turtles. We would like you to consider the following proposal that would, if implemented, lead to the continued protection of these two species of sea turtles:

As part of a nationwide strategy for the continued protection of sea turtles, local utility companies, in collaboration with several conservation agencies, might implement a program to which customers would financially contribute.

The utility companies would use this financial support to donate to organizations that would ensure the preservation of at least 10,000 actively monitored sea turtles.

If the proposal were to pass, funding would come from a monthly, itemized addition to the utility bill of all New York ratepayers (including those who voted against the proposal). Suppose that the money collected will be managed honestly and allocated in full to protecting sea turtles.

The cost of implementing this policy is at present not certain. Because of this we are asking you about several possible cost amounts. When the cost of implementing the proposal is known, the policymaker will be able to look at the votes associated with the actual cost and use this information to help make a policy decision.

What is your vote on the proposal, given that passage of the proposal would cost you these amounts per month? ***(Please indicate a Y or N response for each amount by clicking on the desired response).***

Proposal 6: CLEANUP OF THE GOWANUS CANAL

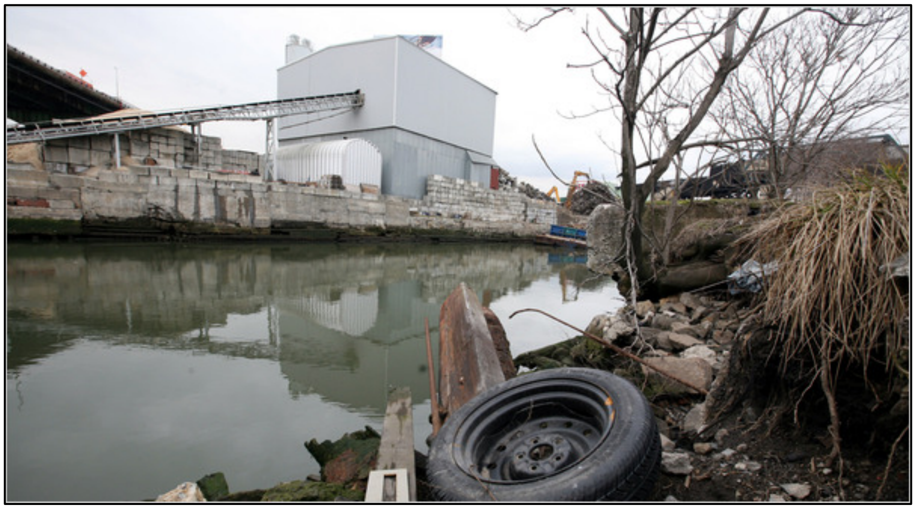

The Gowanus Canal is a canal in the New York City borough of Brooklyn that borders the neighborhoods of Red Hook and South Brooklyn to the west and Park Slope to the east. In 2010 the United States Environmental Protection Agency listed the Gowanus Canal on the National Priority List for cleanup under the federal Superfund law.

Because of design flaws in its construction, the Gowanus Canal does not allow for regular tidal flushing with clean and oxygen-rich water. Water quality studies have found the concentration of oxygen in the canal to be 1.5 parts per million, well below the minimum 4 parts per million needed to sustain life.

With the high level of development in the Gowanus area, large quantities of nitrates and bacteria are constantly flowing into the canal, depleting the oxygen and promoting bacterial growth and odor. Nearby industrial facilities contribute pollutants to the water and sediment, including: oil, mercury, lead, coal tar, and other contaminants. The Gowanus Expressway also passes overhead and deposits pollutants from vehicle emissions into the air and water beneath.

The U.S. Environmental Protection Agency (EPA) has a plan to clean up the canal, which would take about ten years to complete.

Further funding is needed to support the progress of the cleanup. We would like you to consider the following proposal that would, if implemented, lead to the continued remediation of the Gowanus Canal:

As part of New York's strategy for the continued remediation of the Gowanus Canal, local utility companies, in collaboration with the EPA, might implement a program to which customers would financially contribute.

The utility companies would use this financial support to fund the clean up of 10% of the contamination at the Gowanus Canal per year.

If the proposal were to pass, funding would come from a monthly, itemized addition to the utility bill of all New York ratepayers (including those who voted against the proposal). Suppose that the money collected will be managed honestly and allocated in full to remediating the Gowanus Canal.

The cost of implementing this policy is at present not certain. Because of this we are asking you about several possible cost amounts. When the cost of implementing the proposal is known, the policymaker will be able to look at the votes associated with the actual cost and use this information to help make a policy decision.

What is your vote on the proposal, given that passage of the proposal would cost you these amounts per month? *(Please indicate a Y or N response for each amount by clicking on the desired response).*
