# Supplementary material for: The Measurement of Subjective Value and Its Relation to Contingent Valuation and Environmental Public Goods
Source: PLoS One. 2015 Jul 29;10(7):e0132842. doi: 10.1371/journal.pone.0132842 (PMC4519262; doi:10.1371/journal.pone.0132842)
Supplement: S1 File — (DOCX) [file pone.0132842.s002.docx]

Supplementary Methods

**Imaging methods**

We used a 3-Tesla MRI scanner (Siemens Allegra head-only scanner) to measure changes in BOLD activity. During each fMRI scan, a time series of volumes was acquired using a T2*-weighted echo-planar imaging pulse sequence (repetition time, 2000 ms; echo time, 30 ms; flip angle, 75°; thirty-six 3 mm slices with no interslice gap; in-plane resolution, 3 X 3 mm^2^; field of view, 192 mm). Images were acquired using a custom radio frequency coil (NM-011 transmit head coil; Nova Medical Inc.). In addition, T1-weighted high-resolution (1 X 1 X 1 mm^3^) anatomical images were acquired with a magnetization-prepared rapid-acquisition gradient echo pulse sequence and used for across-subjects registration of T2*-weighted images. To minimize head movement, subjects’ heads were stabilized with foam padding. Stimuli were projected onto a screen at the back of the scanner, and subjects viewed them through a mirror attached to the head coil.

**fMRI analysis**

fMRI data were analyzed with the BrainVoyager QX software package (Brain Innovation) and with additional in-house software written in MATLAB (MathWorks Inc.). Preprocessing of functional scans included discarding the first two volumes, slice scan time correction, intersession motion correction (referencing the first volume), and intrasession motion correction (using a subject-specific high-resolution anatomical scan), and the removal of low frequencies up to five cycles per scan (linear trend removal and high-pass filtering). Three sessions from a single subject were removed from analysis for possessing head motion greater than 2 voxels from the reference slice (determined during the scanning session with an online ‘data quality monitor’ provided by the Center for Brain Imaging at New York University). After co-registering each session of each subject with a high-resolution anatomical scan, the images were rotated into the anterior commissure–posterior commissure plane and normalized into Talairach space [1]. For the multi-subject analysis, all images were also spatially smoothed using an 8 mm full-width half maximum Gaussian filter.

**Statistical maps**

Statistical analysis was based on a general linear model [2]. Following the protocol in Levy et al., 2011, the time course of activity of each voxel was modeled with a stick regressor (1 TR in duration) during each trial, convolved with a standard estimate of the hemodynamic impulse response function [3]. For the goods task, the model included a dummy predictor for the first trial of each scan (the first trial of each scan session was always a generic DVD item that was not associated with any valuation procedure included as a “practice” trial, and to account for magnetic saturation effects), a dummy predictor for the presentation of each item, and a parametric predictor of the preference rank for each item, normalized to a 0 – 1 range. No motion regressors were included in any of the linear models in this study. The model was independently fit to each voxel activity time course yielding eight coefficients for each voxel (a nonparametric and parametric regressor for each good category). The threshold for the random-effects maps was set at p < 0.05 (FDR corrected or uncorrected whenever specified). All maps met a default cluster threshold set by Brainvoyager QX of 4 voxels, which yields a spatial extent of at least 100 mm^3^.

Supplementary References

1. Talairach J, Tournoux P. Co-planar Stereotaxic Atlas of the Human Brain, 3-dimensional Proportional System: an Approach to Cerebral Imaging. Stuttgart, 1988.
2. Friston KJ, Holmes, AP, Poline JB, Grasby PJ, Williams SCR, Frackowiak RS, Turner, R. Analysis of fMRI time-series revisited. Neuroimage. 1995; 2(1): 45-53.
3. Boynton GM, Engel, SA, Glover, GH, Heeger, DJ. Linear systems analysis of functional magnetic resonance imaging in human V1. J Neurosci. 1996; 16(13): 4207-4221.
